# The Relationship of Functional Connectivity of the Sensorimotor and Visual Cortical Networks Between Resting and Task States

**DOI:** 10.3389/fnins.2020.592720

**Published:** 2021-01-12

**Authors:** Zhenliang Xiong, Chong Tian, Xianchun Zeng, Jie Huang, Rongpin Wang

**Affiliations:** ^1^Department of Radiology, Guizhou Provincial People’s Hospital, Guiyang, China; ^2^School of Medicine, Guizhou University, Guiyang, China; ^3^Department of Radiology, Michigan State University, East Lansing, MI, United States

**Keywords:** fMRI, functional connectivity, sensorimotor cortex, visual cortex, resting state

## Abstract

The intrinsic activity of the human brain maintains its general operation at rest, and this ongoing spontaneous activity exhibits a high level of spatiotemporally correlated activity among different cortical areas, showing intrinsically organized brain functional connectivity (FC) networks. Many functional network properties of the human brain have been investigated extensively for both rest and task states, but the relationship between these two states has been rarely investigated yet and remains unclear. Comparing well-defined task-specific networks with corresponding intrinsic FC networks may reveal their relationship and improve our understanding of the brain’s operations at both rest and task states. This study investigated the relationship of the sensorimotor and visual cortical FC networks between the resting and task states. The sensorimotor task was to rub right-hand fingers, and the visual task was to open and close eyes, respectively. Our study demonstrated a general relationship of the task-evoked FC network with its corresponding intrinsic FC network, regardless of the tasks. For each task type, the study showed that (1) the intrinsic and task-evoked FC networks shared a common network and the task enhanced the coactivity within that common network compared to the intrinsic activity; (2) some areas within the intrinsic FC network were not activated by the task, and therefore, the task activated only partial but not whole of the intrinsic FC network; and (3) the task activated substantial additional areas outside the intrinsic FC network and therefore recruited more intrinsic FC networks to perform the task.

## Introduction

The brain’s operations are mainly intrinsic, including the acquisition and maintenance of information for interpreting, responding to, and predicting environmental demands (Raichle, 2010). This ongoing intrinsic activity, i.e., the resting-state activity measured with the blood oxygen level dependent (BOLD) functional magnetic resonance imaging (fMRI), is spontaneous but exhibits a high level of spatiotemporally correlated activity among different cortical areas, showing intrinsically organized brain functional connectivity (FC) networks and each network’s temporal coactivity at rest (Ogawa et al., 1992; Biswal et al., 1995; Raichle, 2011). The activity of these FC networks may reflect the brain’s operations at rest, and the study of these FC networks may provide rich and sensitive markers for diseases (Greicius et al., 2004; Filippini et al., 2009). The task fMRI, on the other hand, examines the dynamic brain activity evoked by performing tasks (Kwong et al., 1992; Laird et al., 2013). The activity of neural networks gives rise to simple motor behaviors as well as behaviors that are more complex, and therefore, the activity of a task-specific network is responsible for the specific human behavior. Although the resting-state FC network and the task-specific network reflect two very different cognitive states, i.e., the intrinsic activity vs. the task-evoked activity, these two networks may be related to each other, and studying this relationship may improve our understanding of the brain’s operations at both rest and task states (Cole et al., 2014). Using a novel method, Huang compared the intrinsic activity with task-evoked activity and found that the former was substantially larger than the latter and consistently so for all levels of analysis from a cortical area to the whole brain (Huang, 2019). The study found that, for the task state, the brain (1) controlled the intrinsic activity not only during the performance of a task but also during the rest between tasks; (2) activated a task-specific network only when the task was performed but kept it relatively “silent” for other different tasks; and (3) simultaneously controlled the activation of all task-specific networks during the performance of each task. These results show a strong interaction between the intrinsic activity and task-evoked activity. Understanding this rest-task interaction may be crucial to the elucidation of the brain’s contribution to mental states (Northoff et al., 2010). It may also play an important role in neuroimaging diagnosis and evaluation of neurologically and psychiatrically diseased brains (Castellanos et al., 2013). The study of resting-state fMRI is of great significance for medical imaging diagnosis because it is easy to operate and the scanning process is relatively simple. It only requires the patients to lie down, while the task fMRI requires them to perform tasks, which may not be an easy task for those who have difficulty to carry out the task properly. Nevertheless, neurological and psychiatric diseases may manifest as certain behaviors that may be better characterized by specific task networks such as the face-processing network in Alzheimer’s disease (Huang et al., 2020). Accordingly, interpreting clinical resting-state fMRI data may require a better understanding the relationship of FC network between rest and task states.

Many functional network properties of the human brain have been investigated extensively for both rest and task states, but the relationship between these two states has been rarely investigated yet and remains unclear. The literature shows inconsistent results regarding the relationship between the intrinsic and task-evoked FC networks. Arfanakis et al. (2000) report that the FC, which is demonstrated in the “resting brain,” is not affected by tasks that activate unrelated brain regions. Hampson et al. (2004) found reduced FC between MT/V5 and the cuneus, lingual gyrus, and thalamus but increased FC between MT/V5 and the middle occipital gyrus when viewing moving concentric circles. Fransson and Marrelec (2008) found a global reduction in FC within the default mode network (DMN) during a continuous working memory task. Hasson et al. (2009) reported that the FC network among those regions typically active during rest varies with exogenous processing demands, i.e., the network encompasses more highly interconnected regions during rest than during listening but also when listening to unsurprising vs. surprising information. In comparison to the resting state, Shirer et al. (2012) found increased FC among task-related regions during memory and subtraction tasks. He (2013) found a negative interaction between intrinsic activity and task-evoked activity during a visual attention task. Arbabshirani et al. (2013) reported globally decreased FC networks during the performance of an auditory oddball task. Lynch et al. (2018) also reported reduced FC among different cortical networks, especially between visual and non-visual sensory or motor cortices, when watching a naturalistic movie. Huang (2019) reported a globally greater intrinsic activity compared to the task-evoked activity and the brain’s control to this intrinsic activity not only during the performance of a task but also during the rest between tasks. Cole et al. (2014) suggested that the brain’s functional network architecture during task performance is shaped primarily by an intrinsic network architecture that is also present during rest and secondarily by evoked task-general and task-specific network changes, a strong relationship between intrinsic FC and task-evoked FC. As different methods were used in these studies, their inconsistent results could reflect the FC network difference between the rest and task states or simply were the results of those different methods used for the analyses. To avoid the latter case, in this study, we used the same method to compare the FC networks between the rest and task states.

fMRI-identified FC networks, regardless of rest or task, are determined based on the temporal correlation of the underlying neural activity within each network, reflecting the organized coactivity within that network. The existence of intrinsic FC networks such as the coarse 7-networks and the fine 17-networks is well documented (Yeo et al., 2011). The 7-networks consist of visual, somatomotor, dorsal attention, ventral attention, limbic, frontoparietal, and default networks. The 17-networks further divide these 7 networks into 17 networks. The separation of these networks suggests an organized intrinsic activity within each network, and studying this intrinsic activity for each network may provide insights for understanding the nature of these organized intrinsic activities at rest. On the other hand, fMRI studies of a wide range of sensorimotor, visual, and cognitive tasks reveal simultaneous activation in multiple regions across the whole brain, showing the existence of task-specific networks. Comparing well-defined task-specific networks with corresponding intrinsic FC networks may reveal their relationship. For example, the FC network of the intrinsic somatosensory and motor activity contains both the left and right somatosensory and motor cortices (Yeo et al., 2011). The FC network of tapping the right-hand fingers should, however, contain the left but not the right primary sensory (S1) and motor (M1) cortical areas because the left M1 controls the movement of the right-hand fingers and the left S1 is the primary area for the input of the somatic sensation when tapping these fingers (Penfield and Boldrey, 1937). Accordingly, there should be (1) overlapped or common areas between these two FC networks, (2) areas such as the right S1 and M1 that are present only in the intrinsic FC network, and (3) additional areas outside the intrinsic FC network that are recruited by tapping the right-hand fingers, respectively. To verify this prediction, this study investigated the relationship of the sensorimotor and visual cortical FC networks between the resting and task states.

## Materials and Methods

### Subjects

Eighteen healthy right-handed young adults (10 male and 8 female, ages from 19 to 25 years old) were recruited to participate in this study. Four subjects were excluded from the analysis; three showed substantial head-motion-induced image artifacts and one did not complete the experiment. The Ethics Committee of Guizhou Provincial People’s Hospital approved this study. All subjects consented to participate voluntarily prior to the study. All methods were performed in accordance with the relevant guidelines and regulations of Guizhou Provincial People’s Hospital.

### fMRI Scans

**FIGURE 1:**
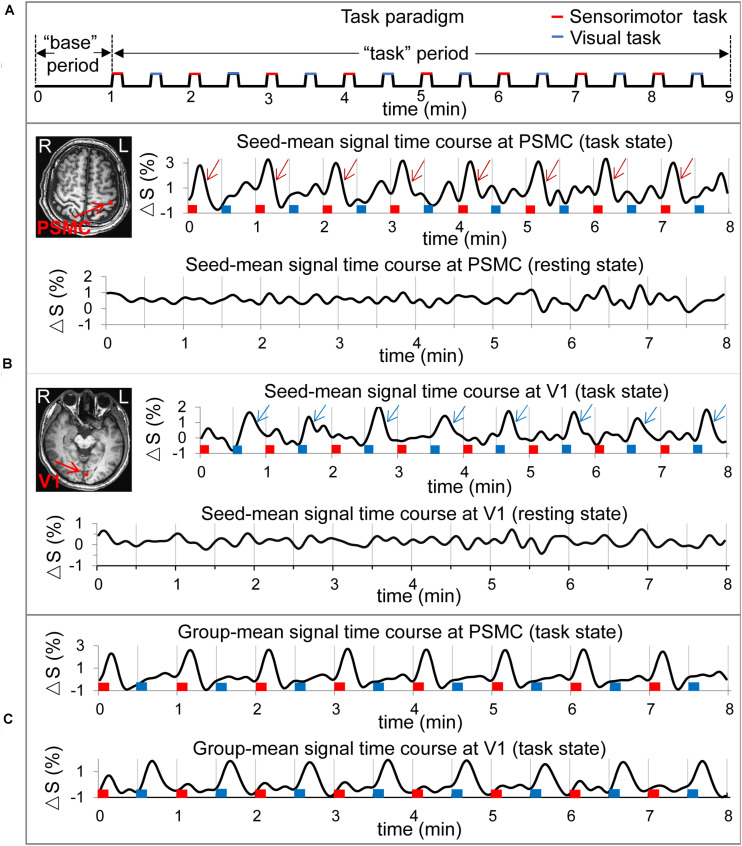
Illustration of the task paradigm, the two selected seeds in the left primary sensorimotor cortex (PSMC) and in the left primary visual area (V1) and their signal time courses of the resting and task states for a representative participant and the group-mean signal change time courses of the selected seeds. **(A)** The task paradigm. The first 1 min served as the “base” period and the last 8 min served as the “task” period. The “task” period consisted of 16 tasks shown by the 16 bars: red bars representing the sensorimotor task and blue bars the visual task. **(B)** Top panel: the red cluster in the left PSMC denotes the seed associated with the finger-rubbing task, and the eight wine red arrows in the right top plot indicate the large signal changes evoked by the eight sensorimotor tasks. The eight red bars represent the onset and duration of the eight finger-rubbing tasks, and the eight blue bars represent the onset and duration of the eight eye-opening and closing tasks, respectively. The right bottom plot illustrates the seed signal time course for the resting state; bottom panel: the red cluster in the left V1 denotes the seed associated with the eye-opening and closing task, and the eight blue arrows in the right top plot indicate the large signal changes evoked by the eight visual tasks. The right bottom plot illustrates the seed signal time course for the resting state. **(C)** Group-mean task-evoked signal change time courses of the selected seeds in the left PSMC and in the left V1, respectively. The task-evoked signal changes are conspicuous for both sensorimotor and visual tasks that are time-locked with these tasks. L, left; R, right.

Each participant first undertook a 9 min resting-state (rs) run and then a 9 min task run. (A dummy scan of 5 volumes prior to each run was discarded). During the rs run, the participants were instructed to close their eyes and try not to think of anything but remain awake for the whole scan. During the task run, they performed two tasks: the first task trial consisted of rubbing five fingers of the right hand for 8 s followed by a 22 s rest period (eyes were closed during the whole trial); and the second task trial consisted of opening eyes for 8 s and then closing them for the 22 s rest period. These two task trials were repeated eight times, resulting in a total of 8 min task period. The participants were instructed to close their eyes during the first 1 min scan, and then, the 8 min task period started (Figure 1A).

### Image Acquisition

MRI data were acquired using a 3.0 T MR scanner (Discovery MR 750, GE Healthcare, Milwaukee, WI) with a 32-channel phased-array coil. Thirty-eight axial T2^∗^-weighted functional images to cover the whole brain were performed using a gradient echo echo-planar-imaging pulse sequence with the parameters: echo time (TE)/repetition time (TR) = 28/2,500 ms, flip angle (FA) = 80°, field of view (FOV) = 224 mm, matrix 64 × 64, slice thickness of 3.5 mm, and spacing of 0.0 mm. Prior to the functional scans, the participants had a pre-training of task performance. They started to rub their fingers when their right legs were tapped twice and stopped the rubbing when the legs were tapped once. When their left legs were tapped twice, they opened their eyes and then closed them when the legs were tapped once. During the task run, these task instructions of tapping the right or left leg were provided by a researcher who stood beside the participant. After the functional scans, T1-weighted whole-brain MR images were performed using a 3D BRAVO pulse sequence.

### Image Pre-processing

Image pre-processing of the functional images was performed with a standard procedure (Huang, 2018), using AFNI^1^. The procedures included the following: (1) removing spikes from the signal intensity time course; (2) slice-timing correction of the image acquisition time difference from slice to slice; (3) motion correction of the images to align all volume images to the first volume image of the rs run; (4) spatial smoothing each volume image with a full-width-half-maximum (FWHM) of 4.0 mm; (5) sorting images for the “base” period (the first 1 min period) and “task” period (the last 8 min period) for each run; (6) computing the mean volume image for both “base” period and “task” period; (7) generating a brain mask with the images of the rs run; (8) bandpassing the signal intensity time course of the “task” period to the range of 0.009–0.08 Hz for both rs and task runs; and (9) computing the relative signal change (%) of the bandpassed signal intensity time course of the “task” period, i.e., relative to the mean signal of the corresponding “base” period, for both rs and task runs. This voxel-wise relative signal change time course of the 8-min “task” period was used to conduct FC analysis for rs and task runs, respectively (Figure 1B), and this ensures the consistency of our FC comparison between the rest and task states.

### Seed Selection and Seed-Dependent Pearson Correlation (R) Maps

We should expect to see (1) a finger-rubbing-induced BOLD signal change in the left primary sensorimotor cortex (PSMC) for each of the eight finger-rubbing tasks and (2) an eye-opening and closing-induced signal change in primary visual cortex (V1) for each of the eight visual tasks. Accordingly, we can examine the signal time courses in these areas to identify one seed in the left PSMC that reflects the time-locked finger-rubbing-induced signal changes and one seed in the area V1 that reflects the time-locked visual stimulation-induced signal changes. For each individual, based on the well-known somatotopic map (i.e., the somatosensory and motor homunculus in PSMC) (Penfield and Boldrey, 1937), we first identified a coarse finger-representation area in the left PSMC. Then, based on the time-locked finger-rubbing-evoked BOLD response, we selected a seed that consisted of four voxels with similar signal change time courses. The same procedure was used to select a seed in V1. For each seed, we computed the mean signal time course, averaged over the four voxels of the seed, for the task run. Then, this mean signal time course was used to compute R with the voxel-wise relative signal change time course of the 8 min “task” period across the whole brain, yielding a seed-dependent R map for the task state. For each participant, two task-associated R maps were generated in the original space, one for the finger-rubbing task and the other for the eye-opening and closing task, respectively. For the resting state, the same two seeds were used to compute the two mean signal time courses in the two cortical areas, similarly as that for the task state. Then, these two time courses were used to generate two R maps for the resting state, one for the sensorimotor network and the other for the visual network, respectively. For group comparison, for each participant, each R map was first warped to a standard template space (icbm452, an averaged volume of 452 normal brains), and then, a mean R map was computed over all participants for that R map, yielding four group-mean R maps corresponding to the two seeds (PSMC vs. V1) and two states (resting vs. task).

### Group Comparison of Seed-Dependent Functional Connectivity Maps Between the Resting and Task States

For each seed and each state, the group-mean R map averaged over all participants was thresholded with a chosen threshold value of *R* = 0.345 (*P* = 1.0 × 10^–6^, *N* = 192) to yield an FC map for that seed and that state. For the seed in the left PSMC, the FC map of the resting state reflects the significant correlation of the intrinsic neural activity at that area with all other cortical areas (i.e., the sensorimotor network at the resting state), and the FC map of the task state shows the finger-rubbing-evoked significant coactivity across the whole brain. Similarly, for the seed in area V1, the FC map of the resting state reflects the significant correlation of the intrinsic neural activity at V1 with all other cortical areas (i.e., the visual network at the resting state), and the FC map of the task state shows the eye-opening and closing-evoked significant coactivity across the whole brain. For both PSMC and V1 areas, we generated a mask of the common area of the two FC maps between the resting and task states to examine the effect of the task on the rs network, and two masks of the difference between the two FC maps, one for the rs FC map excluding the task FC map and the other for the task FC map excluding the rs FC map, to examine the network difference between the two states. Then, for each of the two areas (PSMC vs. V1) and the two states (task vs. rs), each mask was used to compute a mask-mean R within that mask for each subject. These mask-mean *R* values were used for group comparison. For group statistical tests, the *R* values were converted to *Z* values through Fisher’s Z transformation to improve the normality of the distribution.

### Validating the Chosen Threshold R for Determining FC Maps

The determined FC maps were obtained with thresholding their corresponding R maps with *R* = 0.345 (*P* = 1.0 × 10^–6^). Different threshold *R* values would yield different FC maps; a larger threshold would yield a smaller FC map and a smaller threshold would yield a larger FC map, respectively. We chose two different threshold *R-*values of *R* = 0.314 (*P* = 1.0 × 10^–5^) and *R* = 0.374 (*P* = 1.0 × 10^–7^) to test their effect on the relationship of FC between the resting and task states. With each threshold R, we also generated three masks to examine the effect of the task on the rs network and the network difference between the two states for each task type as we did in section “Group Comparison of Seed-Dependent Functional Connectivity Maps Between the Resting and Task States.”

### Validating the Selected Seeds for Determining FC Maps

The determined R maps were obtained with the selected seeds, and different seeds may yield different R maps that affect their corresponding FC maps. To test the potential seed effect on the relationship of FC between the resting and task states, in the original space, we changed the seed size from four to eight voxels to test the seed size effect. Considering that these seeds were selected for each individual and the selection may be biased for the analysis, in the standard template space, we selected two seeds of four voxels each to conduct the FC analysis; one seed was located in the left PSMC [−38 mm (L), −27 mm (P), 55 mm (S), MNI] and the other in area V1 [−6 mm (L), −90 mm (P), 7 mm (S), MNI], respectively. With each seed either in the original space or the standard template space, we also generated three masks to examine the effect of the task on the rs network and the network difference between the two states for each task type as we did in section “Group Comparison of Seed-Dependent Functional Connectivity Maps Between the Resting and Task States.”

## Results

### Seed Selection and Seed-Dependent R Maps

We identified one seed in left PSMC that was associated with the finger-rubbing task and one seed in left V1 associated with the eye-opening and closing task for each participant, and Figure 1B illustrates the two selected seeds in these areas for a representative participant. For each identified seed, a seed-mean signal time course was computed for both resting and task states (Figure 1B). For the task state, for each seed type, a group-mean signal time course averaged over all participants was computed, and its association with that task is conspicuous and time locked for each of the eight task trials (Figure 1C). For each seed type, the seed-mean signal time course was used to compute an R map for each state, yielding a total of four R maps (two seeds and two states) for each individual participant.

### Group Comparison of FC Maps Between the Resting and Task States

**FIGURE 2:**
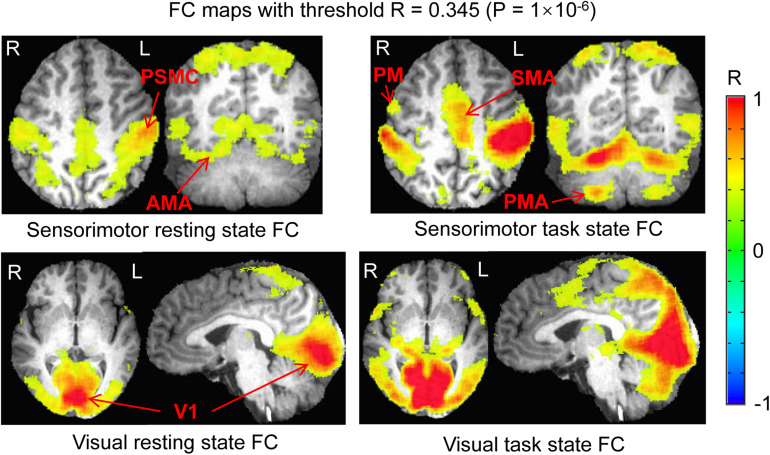
Comparison of the functional connectivity (FC) map between the resting and task states. **Top panel** illustrates the FC map of the resting state that was associated with the intrinsic neural activity of the seed in the left PSMC (left) and the sensorimotor task-evoked FC map across the whole brain (right). **Bottom panel** shows the FC map of the resting state that was associated with the intrinsic neural activity of the seed in the left V1 (left) and the visual stimulation-evoked FC map across the whole brain (right). The color bar indicates the Pearson correlation coefficient R. PSMC, primary sensorimotor cortex; V1, primary visual cortex; SMA, supplementary motor area; PM, premotor area; AMA, anterior motor area; PMA, posterior motor area; L, left; R, right.

**FIGURE 3:**
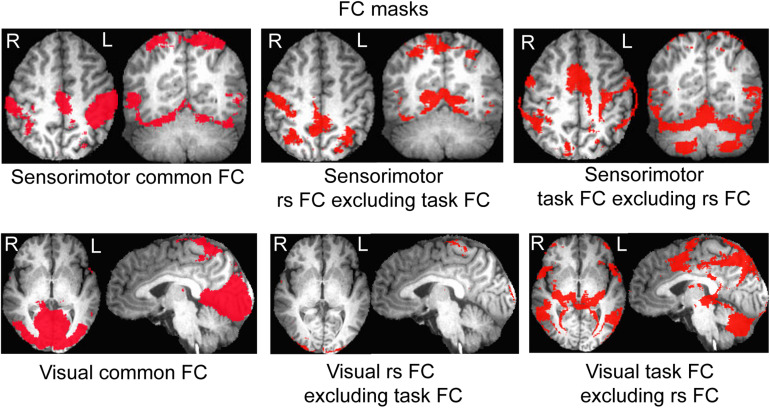
Illustration of the areas of the common and differences of the two functional connectivity (FC) maps between the resting and task states. **Top panel** illustrates the mask of the common FC network of the resting and task states (left), of the areas presented at the resting-state but not the task state (middle), and of the areas presented at the task state but not the resting state (right), respectively, for the sensorimotor system; **bottom panel** shows the mask of the common FC network of the resting and task states (left), of the areas presented at the resting state but not the task state (middle), and of the areas presented at the task state but not the resting state (right), respectively, for the visual system.

**FIGURE 4:**
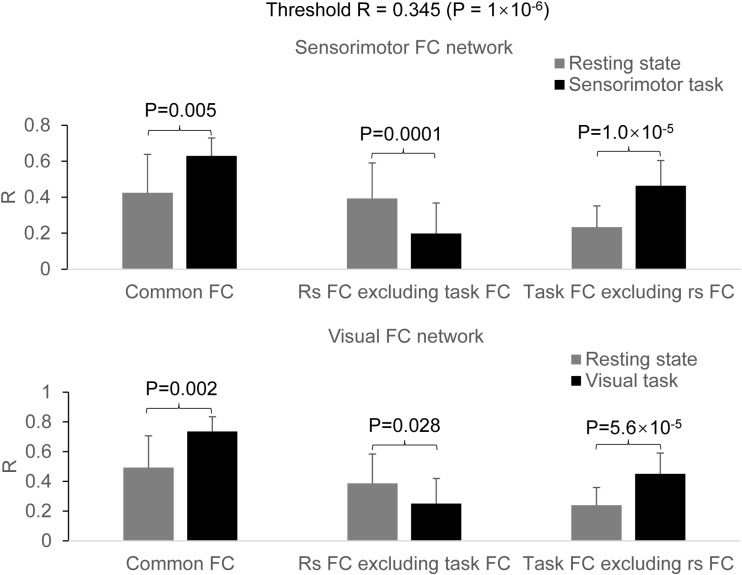
Comparison of the task effect on the functional connectivity (FC) map between the resting and task states. **Top panel**: for the sensorimotor FC map, the finger-rubbing task significantly increased the coactivity across the entire common FC network (two-tail paired *t*-test, *P* = 0.005) (left paired bars) and across those expanded and additionally activated brain areas (two-tail paired *t*-test, *P* = 1.0 × 10^–5^) (right paired bars), respectively. In the resting FC map excluding the task FC map, the R was significantly larger for the resting state than that for the task state (two-tail paired *t*-test, *P* = 0.0001) (middle paired bars); **Bottom panel**: for the visual FC map, the eye-opening and closing task significantly increased the coactivity across the entire common FC network (two-tail paired *t*-test, *P* = 0.002) (left paired bars) and across those expanded and additionally activated brain areas (two-tail paired *t*-test, *P* = 5.6 × 10^– 5^) (right paired bars), respectively. In the resting-state FC map excluding the task FC map, the R was significantly larger for the resting state than that for the task state (two-tail paired *t*-test, *P* = 0.028) (middle paired bars).

For the seed selected in the left PSMC, for the resting state, the determined FC map demonstrated a significant correlation of the intrinsic neural activity in both left and right primary sensorimotor cortex, premotor area, supplementary motor area, parietal cortex, and the right anterior motor area of the cerebellum (Figure 2, top panel). The finger-rubbing task activated not only these regions but also some other areas in cerebrum. The left two images in the top panel of Figure 3 illustrate the overlapped (i.e., common) areas of the FC maps between the resting and task states, the middle two images illustrate the major areas of the rs FC map excluding the task FC map, and the right two images the major areas of the task FC map excluding the rs FC map. Using the common areas of the two FC maps as a mask, a group-mean analysis of the *R* values between the resting and task states yielded a significantly increased R to this common FC map by the finger-rubbing task (Figure 4, top panel, left), demonstrating that the sensorimotor task significantly enhanced the FC of the neural activity of this common sensorimotor system. One of the two major areas of the rs FC map excluding the task FC map was on the right central sulcus, and the other one was located at the posterior part of the supplementary motor area (Figure 3, top panel, middle). Using the corresponding mask, the group-mean analysis of the *R* values showed a significant R for the resting state compared to that of the task state (Figure 4, top panel, middle), demonstrating that the intrinsic neural activity of these areas with that of the seed at the left PSMC was significantly correlated for the resting state, but their neural activity for the task state was not correlated with the sensorimotor task-evoked activity. Comparing to the resting state, the sensorimotor task not only substantially expanded the common FC map but also recruited several additional areas such as both the left and right anterior and posterior motor areas of the cerebellum (the right two images in the top panel of Figure 3). Using these areas as a mask, the group-mean analysis showed a significantly increased R for the task state compared to that for the resting state (Figure 4, top panel, right), demonstrating a significantly expanded task-associated activation network across the whole brain by the finger-rubbing task. To compare the relative size of these three FC maps, i.e., the three FC masks in the top panel of Figure 3, we computed the total number of voxels for each FC mask. Using the total number of voxels of the shared common FC map as the reference, the ratio of the area for the three networks was 1:1.16:2.61 (shared common FC/rs FC excluding task FC/the task FC excluding rs FC). The anatomic locations for each network are tabulated in Table 1.

**TABLE 1:** Brain regions of the common areas shared by both resting state (rs)- and task-functional connectivity (FC) networks, the distinct areas of the rs-FC network from those common areas, and the distinct areas of the task-FC network from those common areas, respectively, for the sensorimotor network labeled in the atlas of TT_Daemon.

**Common areas shared by both rs- and task-FC networks**	**Distinct areas of the rs-FC from those common areas**	**Distinct areas of the task-FC from those common areas**
L&R postcentral gyrus	L&R postcentral gyrus	L&R middle frontal gyrus
L&R inferior parietal lobule	R precuneus	L&R inferior parietal lobule
L&R superior parietal lobule	L&R superior temporal gyrus	L middle frontal gyrus
L&R medial frontal gyrus	L&R cuneus	L&R insula
L&R superior temporal gyrus	L&R middle occipital gyrus	L&R inferior frontal gyrus
L&R middle temporal gyrus	R inferior parietal lobule	L thalamus
L&R inferior temporal gyrus	L lingual gyrus	L&R postcentral gyrus
L declive of vermis	L&R inferior occipital gyrus	R precentral gyrus
L&R declive	R cingulate gyrus	L lentiform nucleus
L&R inferior frontal gyrus	L&R declive	L thalamus
L&R middle occipital gyrus	L inferior occipital gyrus	R superior parietal lobule
L&R precentral gyrus	L&R fusiform gyrus	L&R superior temporal gyrus
L&R fusiform gyrus	L superior pararietal lobule	R middle temporal gyrus
L&R uvula	L precentral gyrus	L&R superior frontal gyrus
L&R parahippocampal gyrus	L culmen of vermis	L fusiform gyrus
L cingulate gyrus	L&R transverse temporal gyrus	L culmen
L lentiform nucleus	L&R tuber	L lentiform nucleus
R cingulate gyrus	L uvula	L putamen
R pulvinar		L&R cerebellar lingual
R lingula gyrus		L&R cerebellar tonsil
L amygdale		L subthalamic nucleus
L thalamus		L&R uvula
R dentate		L&R tuber
R paracentral lobule		L mammillary body
R pyramids		L&R inferior temporal gyrus
L transverse temporal gyrus		L&R inferior semi-lunar lobule
L lateral globus pallidus		L&R pyramis
L insula		L ventral posterior medial nucleus
R fastigium		L&R pyramis of vermis
L medial geniculate body		L&R tuber of vermis
R cerebellar lingual		L parahippocampal gyrus
L subcallosal gyrus		L mammillary body
L putamen		L parahippocampal gyrus
R cerebellar tonsil		L precuneus
R tuber of vermis		L pulvinar
		R declive
		L claustrum
		L dentate
		L red nucleus
		R uvula of vermis
		L substantia nigra

*L, left; R, right.*

For the seed selected in the left V1, for the resting state, the identified FC map showed a significant correlation of the intrinsic neural activity in both the left and right visual cortex (Figure 2, bottom panel). The eye-opening and closing task activated the visual cortex, and this activation extended outside the visual cortex as illustrated in the right images in the bottom panel of Figure 2. The left two images in Figure 3 bottom panel illustrate the mask of the common FC map between the resting and task states, the middle two images illustrate the major areas of the rs FC map excluding the task FC map, and the right two images the major areas of the task FC map excluding the rs FC map, respectively. For the common FC map, a group-mean analysis of the *R* values between the resting and task states showed a significantly increased R for the task state (Figure 4, bottom panel, left), showing that the visual task significantly enhanced the FC within this common FC map compared to the resting state. Using the mask of the major areas of the rs FC map excluding the task FC map (the middle two images in the bottom panel of Figure 3), the group-mean analysis of the *R* values showed a significant R for the resting state compared to that of the task state (Figure 4, bottom panel, middle), demonstrating that the intrinsic neural activity of these areas with that of the seed at the left V1 was significantly correlated for the resting state, but their neural activity for the task state was not correlated with the visual task-evoked activity. For those areas of the task FC map excluding the rs FC map (the right two images in the bottom panel of Figure 3), the group-mean analysis showed a significantly increased R for the task state compared to that for the resting state (Figure 4, bottom panel, right), demonstrating a significantly expanded task-associated activation network across the whole brain by the eye-opening and closing task. To compare the relative size of these three FC maps, i.e., the three FC masks in the bottom panel of Figure 3, we computed the total number of voxels for each FC mask. Using the total number of voxels of the shared common FC map as the reference, the ratio of the area for the three networks was 1:0.08:1.85 (shared common FC/rs FC excluding task FC/the task FC excluding rs FC). The anatomic locations for each network are tabulated in Table 2.

**TABLE 2:** Brain regions of the common areas shared by both rs- and task-functional connectivity (FC) networks, the distinct areas of the rs-FC network from those common areas, and the distinct areas of the task-FC network from those common areas, respectively, for the visual network labeled in the atlas of TT_Daemon.

**Common areas shared by both rs- and task-FC networks**	**Distinct areas of the rs-FC from those common areas**	**Distinct areas of the task-FC from those common areas**
L & R lingual gyrus	L&R postcentral gyrus	L cingulate gyrus
L & R inferior occipital gyrus	R middle occipital	L&R superior temporal gyrus
L middle occipital gyrus	L&R culmen	R inferior temporal gyrus
L & R middle temporal gyrus	L&R inferior parietal lobule	R superior frontal gyrus
R middle occipital gyrus	L&R medial frontal gyrus	L&R middle temporal gyrus
L & R cuneus	L&R precuneus	L&R precentral gyrus
L culmen	L&R declive	L&R middle frontal gyrus
L & R declive	L&R paracentral lobule	R middle occipital gyrus
L&R uvula	R lingual gyrus	R posterior cingulate
L & R postcentral gyrus	R superior parietal lobule	L&R fusiform gyrus
R superior temporal gyrus	L postcentral area	L cerebellar lingual
L & R fusiform gyrus	left cuntate	L&R culmen
L&R superior parietal lobule	R Brodmann area 37	L&R hippocampus
R posterior cingulated	L precentral gyrus	L inferior frontal gyrus
L&R transverse temporal gyrus	R cuneus	R medial geniculate body
L precuneus	R dentate	L&R parahippocampal gyrus
L&R pyramis		L&R uvula
L&R tuber		L&R declive
		L&R inferior semi-lunar lobule
		L&R cerebellar tonsil
		L&R pyramis
		L&R pulvinar
		L&R thalamus
		R inferior occipital gyrus
		R uvula of vermis
		L lentiform nucleus
		R lingual gyrus
		L tuber
		L caudate
		L claustrum
		L dentate
		L transverse temporal gyrus
		R vermis
		L insula

*L, left; R, right.*

### Validating the Chosen Threshold R for Determining FC Maps

**FIGURE 5:**
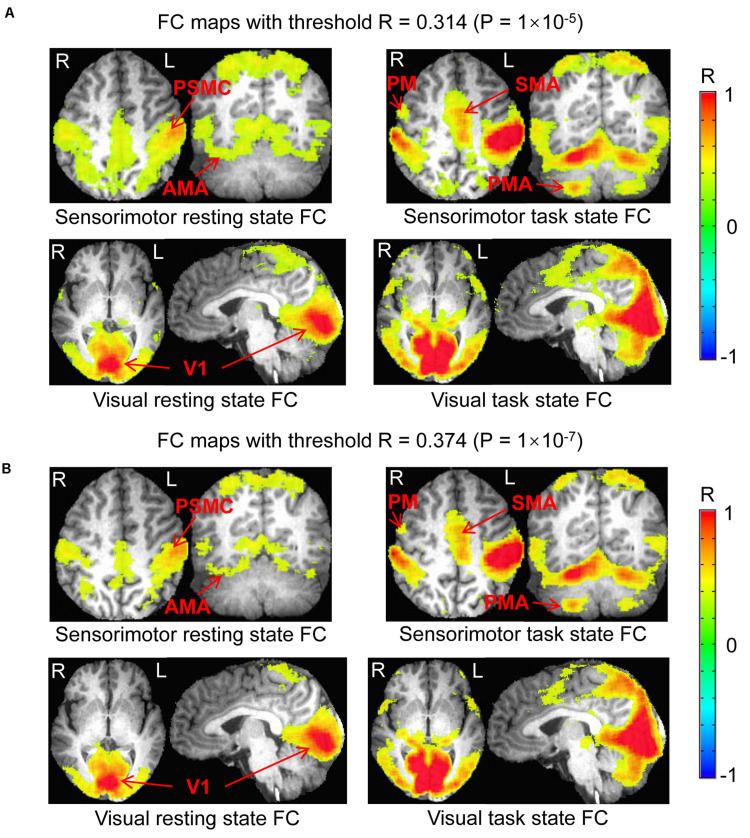
Comparison of the functional connectivity (FC) map between the resting and task states determined with the two different threshold *P* = 1.0 × 10^– 5^ and 1.0 × 10^– 7^. **(A)** Top panel illustrates the sensorimotor FC map of the resting state vs. task state, and the **(B)** bottom panel shows the visual FC map of the resting state vs. task state, respectively. PSMC, primary sensorimotor cortex; V1, primary visual cortex; SMA, supplementary motor area; PM, premotor area; AMA, anterior motor area; PMA, posterior motor area; L, left; R, right.

**FIGURE 6:**
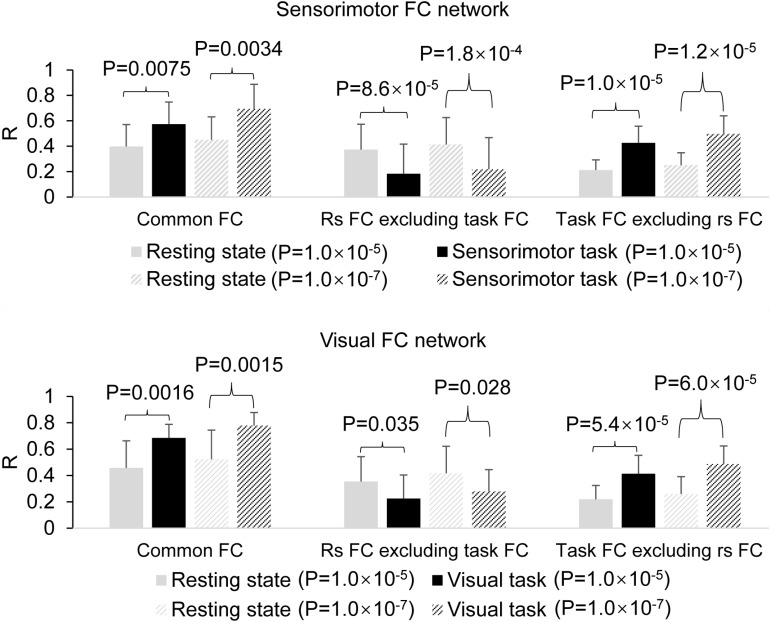
Comparison of the task effect on the functional connectivity (FC) map between the resting and task states determined with the two different threshold P = 1.0 × 10^–5^ and 1.0 × 10^–7^. The **top panel** illustrates the effects of the finger-rubbing task on the sensorimotor FC map, and the **bottom panel** illustrates the effects of the eye-opening and closing task on the visual FC map.

The determined FC maps with the two different threshold *P* = 1.0 × 10^–5^ and 1.0 × 10^–7^ demonstrated almost the same FC networks as that determined with *P* = 1.0 × 10^–6^ for both resting and task states (Figure 5), showing that the general pattern of these FC networks held despite different threshold R (*P*) values. With the generated three masks (images not presented) for each threshold *P*-value, similar results were obtained (Figure 6), showing that these two different threshold *P*-values produced the same relationship of FC between the resting and task states.

### Validating the Selected Seeds for Determining FC Maps

**FIGURE 7:**
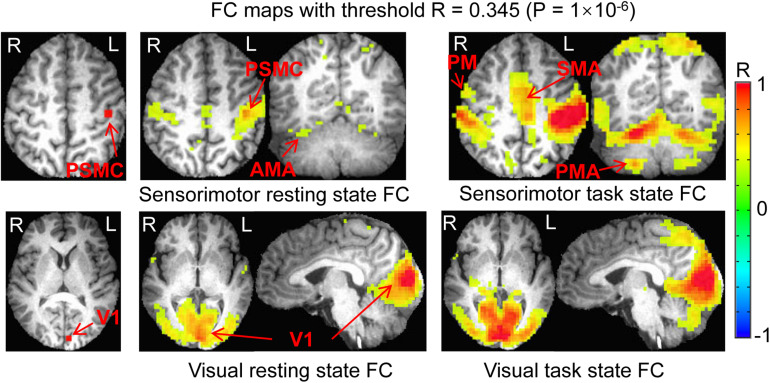
Comparison of the functional connectivity (FC) map between the resting and task states determined with the two seeds selected in the standard template space. **Top panel** illustrates the seed selected in the left PSMC and the sensorimotor FC map of the resting state vs. task state. **Bottom panel** shows the seed selected in the left V1 and the visual FC map of the resting state vs. task state. PSMC, primary sensorimotor cortex; V1, primary visual cortex; SMA, supplementary motor area; PM, premotor area; AMA, anterior motor area; PMA, posterior motor area; L, left; R, right.

**FIGURE 8:**
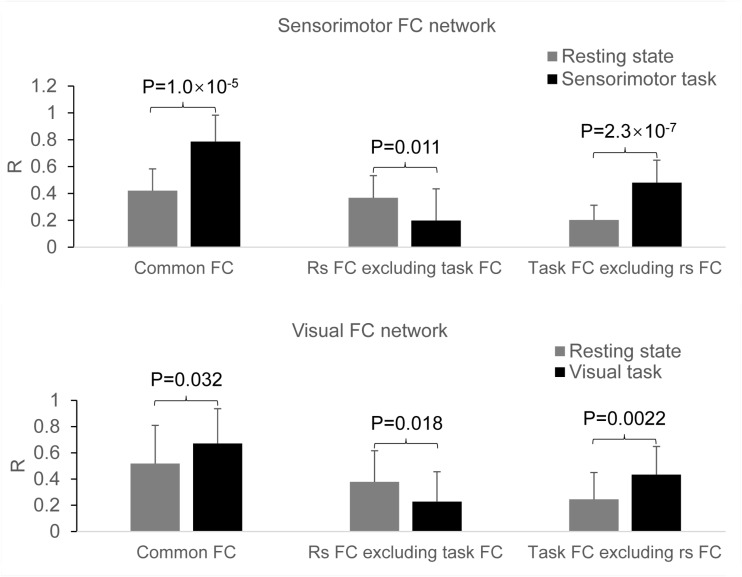
Comparison of the task effect on the functional connectivity (FC) map between the resting and task states determined with the two seeds selected in the standard template space. **Top panel**: the effects of the finger-rubbing task on the sensorimotor FC map; **bottom panel**: the effects of the eye-opening and closing task on the visual FC map.

In the original space, the selected two seeds with eight voxels each produced almost identical results as those with four voxels each (data not presented). In the standard template space, the selected two seeds produced similar FC networks as those obtained with the selected two seeds in the original space (Figure 7). With the generated three masks (images not presented), comparing the rest FC with the task FC showed the same relationship of FC between the resting and task states (Figure 8).

## Discussion and Conclusion

This study investigated the relationship of the sensorimotor FC network between the resting and the task state of rubbing the fingers of the right hand. The results verified our prediction that these two FC networks are related in a specific way. First, they share a common FC network as shown in Figure 3 (top panel, left). Second, as expected, the right M1 and S1 areas are not present in the task-evoked FC network (Figure 3, top panel, middle). Third, the performance of this finger-rubbing task recruited, outside the intrinsic FC network, substantial areas across both cerebrum and cerebellum (Figure 3, top panel, right). These results do not support the suggestion that the brain’s functional network architecture during task performance is shaped primarily by an intrinsic network architecture that is also present during rest and secondarily by evoked task-general and task-specific network changes (Cole et al., 2014). These substantial additional areas recruited by the task performance indicate the involvement of other intrinsic FC networks when performing the task, showing a complicated relationship of this task-evoked FC network with those intrinsic FC networks. As the task is a simple sensorimotor task, we also expect a complicated relationship between intrinsic and task-evoked FC networks when performing complex tasks.

The shared common FC network (Figure 3, top panel, left) shows a significantly increased R for the task state than that for the resting state (paired *t*-test, *P* = 0.005) (Figure 4, top panel, left), showing that the task performance significantly enhances the co-activity within that network compared to the intrinsic neural activity at rest. This conclusion is also illustrated in the top panel in Figure 2. This common FC network may be an essential part for the finger-rubbing task. The control of the left primary motor cortex of the cerebrum to the movement of the fingers of the right-hand and similarly the control of the right primary motor cortex to the left-hand fingers, i.e., the somatomotor representations, are well documented (Penfield and Boldrey, 1937). The somatosensory representations of the input of sensory information of the right-hand to the left primary sensory cortex and the input of the sensory information of the left-hand to the right primary sensory cortex, respectively, are also well documented. The exclusion of the right M1 and S1 areas from the right-hand finger-rubbing-evoked FC network reflects these somatomotor and somatosensory representations (Figure 3, top panel, middle). The important role of the cerebellum in movement control, and the decussate cerebrocerebellar circuit, i.e., the right cerebellar cortex is connected to the left cerebral cortex and the left cerebellar cortex is connected to the right cerebral cortex, respectively, is also well documented. This cerebrocerebellar circuit mediates a two-way connection between the cerebrum and cerebellum and plays a crucial role in somatic functions concerning motor planning, motor coordination, motor learning, and memory (Allen and Tsukahara, 1974; Benagiano et al., 2018). Right-hand finger rubbing activates not only the contralateral cerebrocerebellar circuit with respect to the cerebrum but also the ipsilateral cerebrocerebellar circuit as evidenced in the right images in the top panel of Figure 2, showing an association between these two circuits and a complicated task-evoked FC network even for a simple finger-rubbing task. The contralateral cerebrocerebellar circuit consists of M1, premotor and supplementary motor areas in the left cerebrum and both anterior and posterior motor areas in the right cerebellum and is mainly responsible for the motor planning, coordination, and execution of rubbing the fingers of the right hand. The ipsilateral cerebrocerebellar circuit, however, consists of premotor and supplementary motor areas in the right cerebrum and both anterior and posterior motor areas in the left cerebellum, i.e., excluding the right M1 area compared to the contralateral cerebrocerebellar circuit, and its functional role in the performance of rubbing right-hand fingers is unknown. These results replicate those previous findings (Huang, 2020). Further studies are needed to explore the functional role of this ipsilateral cerebrocerebellar circuit.

This study also investigated the relationship of the visual FC network between the resting and the task state of opening and closing eyes. The results demonstrate a similar relationship as that of the sensorimotor FC network between the resting state and the finger-rubbing task state: (1) they share a common FC network as shown in Figure 3 (bottom panel, left); (2) a few areas both inside and outside the visual cortex are present only in the intrinsic FC network (Figure 3, bottom panel, middle); and (3) substantial areas outside the intrinsic FC network are recruited by the opening and closing eyes (Figure 3, bottom panel, right). The shared common FC network shows a significantly increased R for the task state than that for the resting state (paired *t*-test, *P* = 0.002) (Figure 4, bottom panel, left), showing that opening and closing eyes significantly enhances the coactivity within that network compared to the intrinsic neural activity at rest. This common FC network is mainly in the visual cortex, and the bottom panel in Figure 2 illustrates the task-enhanced coactivity within that network. In comparison to the intrinsic neural activity at rest, the substantial additional areas recruited by opening and closing eyes locate mainly outside the visual cortex and extend to the cerebellum as well (Figure 4, bottom panel, right), indicating the involvement of other intrinsic FC networks when performing this task. It shows a complicated relationship of this eye-opening- and eye-closing-evoked FC network with intrinsic FC networks, a conclusion similar as that of the finger-rubbing-evoked FC network.

The group-mean R in the shared common FC network was significantly larger for the task than that for the rest (Figure 4, left), regardless of the task type, showing a task-enhanced coactivity within the network in comparison to the intrinsic activity. In contrast to the task paradigm of 8-s task on followed by 22 s task off for each of the 16 task trials, our recent study with a continuous alternating 2 s visual stimulation on-and-off task paradigm observed a similar task-enhanced coactivity in the visual FC (Huang and Zhu, 2017), showing that this task-enhanced coactivity is independent of the task paradigms. The intrinsic activity was irregular, spontaneous, and self-regulated, but the task-evoked activity was actively controlled by the brain, reflected in the task-fMRI time series that was regular and time locked to the task paradigm (Figure 1B). This regularity and time-locked behavior were the results of the brain’s actively controlling the task performance and therefore should reflect the underlying neuronal activity evoked by performing the task. In comparison to the intrinsic activity, the task-enhanced coactivity in the common FC network shows a stronger effect of the brain’s active control to the task-evoked activity. It reflects a different degree of the brain’s control to these two different brain states, i.e., the self-regulated intrinsic activity in the resting state vs. brain’s actively controlled task-evoked neuronal activity in the task state. Our recent study demonstrates the brain’s active control to the intrinsic activity during the task state (Huang, 2019). The study systematically compared the intrinsic activity with the task-evoked activity at several levels starting from a finger-tapping-activated area in the PSMC, then the task-activated areas across the whole brain, and finally the gray matter, white matter, and whole brain. At each level, the intrinsic activity was found to be equal to or substantially larger than the task-evoked activity. The study also found that the brain substantially suppressed the intrinsic activity not only during the period of task performance but also during the rest period between the tasks, reflecting the brain’s active control to the intrinsic activity during the task state.

This study also found that changing seed size (four vs. eight voxels) and selecting seeds in the original space for each individual subject vs. common seeds in the standard template space for all subjects produced similar results for both rest FC and task FC (Figures 5–8), showing that the relationship of FC of the sensorimotor and visual networks between the resting and task states remained unchanged under these conditions.

In conclusion, this study shows a general relationship of a task-evoked FC network with its corresponding intrinsic FC network, regardless of tasks. For each task type, the study shows that (1) the intrinsic and task-evoked FC networks share a common network and the task enhances the coactivity within that common network compared to the intrinsic activity; (2) some areas within the intrinsic FC network are not activated by the task, and therefore, the task activates partial but not whole of the intrinsic network; and (3) the task activates substantial additional areas outside the intrinsic FC network and therefore recruits more intrinsic FC networks for the task performance.

## Data Availability Statement

The original contributions presented in the study are included in the article/supplementary material, further inquiries can be directed to the corresponding author/s.

## Ethics Statement

The studies involving human participants were reviewed and approved by the Ethic Committee of Guizhou Provincial People’s Hospital (Guizhou Provincial People’s Hospital). The patients/participants provided their written informed consent to participate in this study.

## Author Contributions

ZX was the guarantor of integrity of the entire study. All authors contributed to the study concepts and design, data analysis and interpretation, and manuscript drafting or manuscript revision for important intellectual content, gave approval of the final version of the submitted manuscript.

## Conflict of Interest

The authors declare that the research was conducted in the absence of any commercial or financial relationships that could be construed as a potential conflict of interest.

## Footnotes

1 http://afni.nimh.nih.gov/afni
